# In Situ Electroporation on PERFECT Filter for High-Efficiency and High-Viability Tumor Cell Labeling

**DOI:** 10.3390/mi13050672

**Published:** 2022-04-26

**Authors:** Tingting Hun, Yi Zhang, Qingmei Xu, Dong Huang, Qi Wang, Zhihong Li, Wei Wang

**Affiliations:** 1 School of Integrated Circuits, Peking University, Beijing 100871, China; huntingting@pku.edu.cn (T.H.); zhangyi21@pku.edu.cn (Y.Z.); qmxu@stu.pku.edu.cn (Q.X.); superhd@pku.edu.cn (D.H.); zhhli@pku.edu.cn (Z.L.); 2 National Key Laboratory of Science and Technology on Micro/Nano Fabrication, Beijing 100871, China; 3 Department of Respiratory Medicine, The Second Affiliated Hospital of Dalian Medical University, Dalian 116027, China; wqdlmu@163.com; 4 Frontiers Science Center for Nano-Optoelectronics, Peking University, Beijing 100871, China

**Keywords:** in situ electroporation, circulating tumor cell, high viability

## Abstract

Labeling-assisted visualization is a powerful strategy to track circulating tumor cells (CTCs) for mechanism study (e.g., tumor metastasis). Due to the rarity of CTCs in the whole blood, efficient simultaneous enrichment and labeling of CTCs are needed. Hereby, novel in situ electroporation on a previously-developed micropore-arrayed filter (PERFECT filter) is proposed. Benefiting from the ultra-small-thickness and high-porosity of the filter plus high precision pore diameter, target rare tumor cells were enriched with less damage and uniform size distribution, contributing to enhanced molecular delivery efficiency and cell viability in the downstream electroporation. Various biomolecules (e.g., small molecule dyes, plasmids, and functional proteins) were used to verify this in situ electroporation system. High labeling efficiency (74.08 ± 2.94%) and high viability (81.15 ± 3.04%, verified via live/dead staining) were achieved by optimizing the parameters of electric field strength and pulse number, ensuring the labeled tumor cells can be used for further culture and down-stream analysis. In addition, high specificity (99.03 ± 1.67%) probing of tumor cells was further achieved by introducing fluorescent dye-conjugated antibodies into target cells. The whole procedure, including cell separation and electroporation, can be finished quickly (<10 min). The proposed in situ electroporation on the PERFECT filter system has great potential to track CTCs for tumor metastasis studies.

## 1. Introduction

The ability to introduce foreign molecules, such as DNA, mRNA, fluorescent dyes, and proteins, into living cells has significant implications for various applications in cellular manipulation [1], cellular imaging [2], genome engineering [3], and disease treatment [4]. Various methods, including viral-mediated, chemical-mediated, and physical-mediated approaches, have been developed to deliver molecules into the cells. The viral-mediated approach is the most popular method of gene delivery because it is highly efficient, while the application remains a considerable safety concern, including toxicity, immunogenicity, and chromosomal integration [5,6,7]. The chemical-mediated approach is often used to deliver intracellular biomolecules, but it also struggles with the toxic effects, particularly molecules and limited cell types [6]. Compared to other physical methods (e.g., microinjection, optoporation, and sonoporation), electroporation is considered an effective and powerful technique because it is easier to perform and has the ability to introduce types of molecules into target cells without potentially damaging cells [8,9].

Electroporation utilizes short and high voltage pulses to cause a temporary loss of the semipermeability of cell membranes and delivers molecular probes into the cytosol [10]. It is widely accepted that hydrophilic pores, electrically altered lipids, and modulated voltage-gated ion channels increase membrane permeability within microseconds [11,12,13]. Technically, the efficiency of intracellular delivery can be optimized by modulating the pulse duration, frequency, and voltage. Besides, the electric field strength required to transiently disrupt cellular membranes is strongly correlated with cell size [14]. In conventional electroporation designs, bulk electroporation has been used for effective DNA transfection into suspension cells [15]. However, in order to obtain practical efficiency and viability for samples with large heterogeneity in cell diameter, a strong electric field was used, which might lead to the destruction of the cell membrane [16]. When evaluating the performance of electroporation, the following points should be considered: (1) electroporation efficiency and cell viability with an easy operation; (2) controllability at the single-cell level; and (3) selectivity between targeted cells and background cells (e.g., rare circulating tumor cells (CTCs) and blood cells).

A series of researchers have explored various nano-devices and micro-devices to operate single-cell electroporation, which effectively decreases the applied voltage, resulting in enhanced cell viability [17,18,19,20,21,22,23]. The nano-devices, including nanochannel [19,24], nanopillar [25], nanostraw [26,27], nanoprobe [28], nanopore [29,30], nanoelectrode [31], etc., are focused on a nanosized portion of the cell membrane, which enables the efficient and precise amount of molecule delivery [17]. However, the aforementioned methods suffer from relatively low throughputs. Meanwhile, micro-devices mainly include microfluidic channels [32,33], microcapillaries [34], microarrays [35,36,37], etc., which make it easier to achieve high-throughput paralleled transfection. However, additional assistance must be operated for cell localization, such as using microfluidic devices to squeeze cells or vacuum set-ups to trap cells, which may affect the viability of target cells. No reports have yet realized high-throughput CTC enrichment and labeling simultaneously in nano/microdevices, which could be attributed to the poor cell viability and failure in effective electroporation caused by large forces (shear stress, pressure) endured during separation.

**Figure 1:**
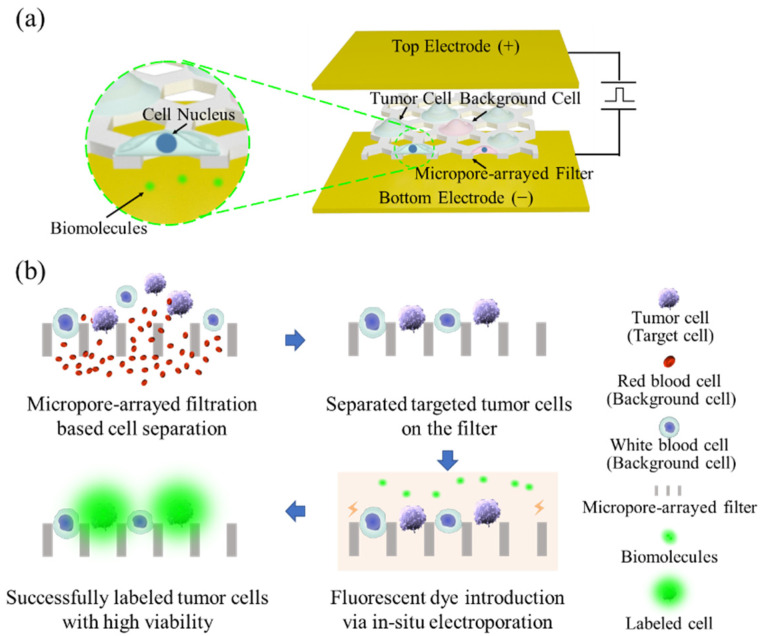
A schematic representation (**a**) and workflow overview (**b**) of the in situ electroporation system on a PERFECT filter.

In our previous study, a PERFECT (precise, efficient, rapid, flexible, easy-to-operate, controllable, and thin) filter with high porosity and high mechanical strength was prepared via the Parylene C molding technique. Benefiting from the ultra-small-thickness (<10 μm) and high-porosity (46.79%, edge-to-edge space between the adjacent micropores <4 μm) of the filter plus the high precision pore diameter, this filter significantly decreases the forces applied on cells during filtration [38] and thus ensures high cell viability, which facilitates the feasibility of electroporation on separated cells. In this study, in situ electroporation on the PERFECT filter was proposed to realize high-throughput, high-efficiency, and high-viability tumor cell separation and electroporation simultaneously (Figure 1). Multiple molecules have been delivered to verify the excellent performance of this device, which may provide a new tool for CTC tracing in vivo.

## 2. Materials and Methods

### 2.1. Set-Up of In Situ Electroporation System

The micropore-arrayed filter with ultra-small-thickness (<10 μm) and high-porosity (46.79%)-based tumor cell separation builds on our previous work [39]. The optimum micropore diameters (4 μm space and 10 μm diameter) for cell viability under the electric field strengths and shear stresses were also preliminarily explored [40]. After separation, the target tumor cells were captured on the filter, and transferred into a commercially-available cuvette (620, 2 mm Gap Cuvette, BTX) with two parallel-plate electrodes for in situ electroporation. A modified electroporation buffer (13 mM KH_2_PO_4_, 13 mM K_2_HPO_4_, 25 mM myo-inositol) was used for this in situ electroporation. The magnitude of applied square wave pulses, V, was varied from 100 to 400 V to have the electric field strength, E = V/L (L: 2 mm), applied across the electroporation chambers ranging from 0.5 to 2 kV/cm. The applied magnitude, pulse duration (100 μs, 1000 μs, 3000 μs, 5000 μs, 10,000 μs), and pulse number (3 and 6) were optimized respectively to determine the optimum electrical condition for multiple molecule delivery.

### 2.2. Cell Culture and Counting

Mouse lung cancer cells (1601), separated and prepared by the collaborator, were used as a model cell in this study [41,42]. The 1601 cells were cultured with high glucose Dulbecco’s modified Eagle medium (DMEM-high glucose, Corning, New York, NY, USA) supplemented with 10% fetal bovine serum (FBS, Gibco, Thermo Fisher, Waltham, MA, USA), 2 mM glutamine (Gibco, Thermo Fisher, USA), 1 mM sodium pyruvate (Gibco, Thermo Fisher, USA), 25 mM HEPES (Gibco, Thermo Fisher, USA), and 100 U/mL penicillin-streptomycin (Gibco, Thermo Fisher, USA). Cells were incubated at 37 °C in a 5% CO_2_ humidified atmosphere. When the confluency reached 80–90%, cells were trypsinized from the flask, centrifuged at 1000 rpm for 6 min, then resuspended in DMEM at a concentration of 5 × 10^5^ cells/mL for separation and electroporation.

To verify the separation and electroporation rate of presented in situ electroporation systems, spiking PBS or undiluted whole blood from healthy volunteers (with the statement of informed consent) were tested first. The spiking samples were prepared by adding 10,000 1601 cells via serial dilutions into 1 mL PBS or whole blood. Before adding, the adhered 1601 cells were rinsed with trypsin (0.25%, Gibco, Thermo Fisher, USA) and then incubated with 5 μg/mL Hoechst 33342 (H1399, Invitrogen™, Thermo Fisher, USA) and Cell Tracker Red/Green (5 μM, Invitrogen, Thermo Fisher, USA) at room temperature for 30 min for pre-labeling. After incubation, the cells were washed with PBS 3 times and resuspended into the PBS or blood. Note that pre-labeling was operated to facilitate the observation and counting of 1601 among background cells under the fluorescence microscope.

### 2.3. Fluorescent Molecules Used for Device Optimization

The nucleic acid fluorescent dyes (4 µM, EthD-1, Invitrogen™, Thermo Fisher, USA) were used to access the current system’s small molecule delivery efficiency. The choice of fluorescent dye was made because of its impermeability for live cells, and thus can only be loaded into cells under successful electroporation. Therefore, it can be used to access the electroporation efficiency of the presented device in this work. In order to identify the cell viability, fluorescent live-cell staining dyes (2 µM, Calcein Green AM, Invitrogen™/Thermo Fisher, USA) were selectively performed before and after electroporation. The double-positive red and green fluorescence (i.e., EthD-1+/Calcein Green AM+) stands for successful electroporation and cell viability. The positive for green while negative for red fluorescence (i.e., Calcein Green AM+/EthD-1-) stands for cells with viability and unsuccessful electroporation. The positive for red while negative for green fluorescence (i.e., Calcein Green AM-/EthD-1+) stands for cell death.

The anti-cytokeratin 7 (CK7) fluorescent dye-conjugated antibody (ab209601, Abcam, Boston, MA, USA) (1:200 dilution, rabbit-anti-mouse, Alexa Fluor^®^ 555) was used to access the current system’s delivery efficiency of proteins. In this work, the antibody was directly mixed with electroporation buffer. Then, target cells were separated on the filter and placed into the buffer for in situ electroporation. Subsequently, electroporation efficiency was calculated by counting the number of fluorescent-labeled cells and the number of total target cells. The whole procedure, including cell separation and electroporation, can be finished within 10 min.

### 2.4. Traditional and Improved Immunofluorescence Staining

The traditional immunofluorescence staining can achieve specific labeling of CTCs through a series of operations, but fails to maintain the viability of target cells. After filtration, the cells on the membranes were fixed with 4% (*w*/*v*) paraformaldehyde for 10 min, followed by washing three times with PBS for 5 min each. Next, permeabilized with 0.1% (*v*/*v*) Triton X-100 for 7 min at room temperature, followed by washing three times with PBS for 5 min each. Then, the cells were blocked with blocking buffer for 60 min at room temperature. After blocking nonspecific binding sites, cells were incubated with anti-cytokeratin 7 (CK7) fluorescent dye-conjugated antibody (ab209601, Abcam, USA) at 4 °C overnight. In order to improve the viability of labeled cells and reduce the staining time, we improved the traditional immunofluorescence staining method. The improved immunofluorescence staining contains cell permeabilized with 0.1% (*v*/*v*) Triton X-100 for 3 min at room temperature, followed by washing three times with PBS for 5 min each and incubated with anti-cytokeratin 7 (CK7) fluorescent dye-conjugated antibody 10 min at room temperature.

### 2.5. Data Analysis

In the delivery experiment of small molecule dyes, the living cells were stained with Calcein Green AM (green fluorescent dye), and the transfected cells were successfully stained with EthD-1 (red fluorescent dye). In the delivery experiment of plasmids, the live/dead staining was used for cell viability validation (the dead cells were stained with red fluorescent dye), and the transfected cells were successfully transfected with a plasmid containing green fluorescent protein (GFP). In the delivery experiment of dye-conjugated antibodies, the live/dead staining was also used for cell viability validation (the living cells were stained with green fluorescent dye), and the tumor cell was specifically labeled with red fluorescent when the electroporation was successful.

The cell viability, transfection efficiency after electroporation, and total cell viability after filtration and electroporation were calculated based on the following equations:

$$\mathrm{Cell}\;\mathrm{viability}\;\left(\mathrm{electroporation}\right)=\frac{\mathrm{The}\;\mathrm{number}\;\mathrm{of}\;\mathrm{living}\;\mathrm{cells}\;\mathrm{on}\;\mathrm{the}\;\mathrm{filter}}{\mathrm{The}\;\mathrm{number}\;\mathrm{of}\;\mathrm{total}\;\mathrm{cells}\;\mathrm{on}\;\mathrm{the}\;\mathrm{filter}}\times 100\%$$

$$\mathrm{Electroporation}\;\mathrm{efficiency}\;=\frac{\mathrm{The}\;\mathrm{number}\;\mathrm{of}\;\mathrm{transfected}\;\mathrm{cells}}{\mathrm{The}\;\mathrm{number}\;\mathrm{of}\;\mathrm{total}\;\mathrm{cells}\;\mathrm{on}\;\mathrm{the}\;\mathrm{filter}}\times 100\%$$

$$\mathrm{Cell}\;\mathrm{viability}\;\left(\mathrm{total}\right)=\frac{\mathrm{The}\;\mathrm{number}\;\mathrm{of}\;\mathrm{living}\;\mathrm{cells}\;\mathrm{on}\;\mathrm{the}\;\mathrm{filter}}{\mathrm{The}\;\mathrm{number}\;\mathrm{of}\;\mathrm{total}\;\mathrm{spiked}\;\mathrm{cells}\;}\times 100\%$$

## 3. Results and Discussion

### 3.1. Optimum Electrical Parameters for High-Efficiency Small Molecule Delivery

According to our previous study [40], the filter with 4 μm space and 10 μm diameter with a porosity of 46.79% was chosen in this work. First, the recoveries of 10,000 1601 cells spiked in PBS (1 mL) or undiluted whole blood (1 mL) showed high recovery rates (79.3 ± 2.5%, 76.3 ± 5.9%, respectively, *n* = 3 for every trial) and high cell viability (86.0 ± 5.6%, 83.0 ± 8.7%, respectively, *n* = 3 for every trial) indicating the high separation performance of this platform (see Supplementary Material Figure S1). After cell separation, A series of electroporation experiments with systematically varied electric field strengths and pulse numbers were conducted in order to determine the optimum condition for high-efficiency electroporation of 1601 cells.

**Figure 2:**
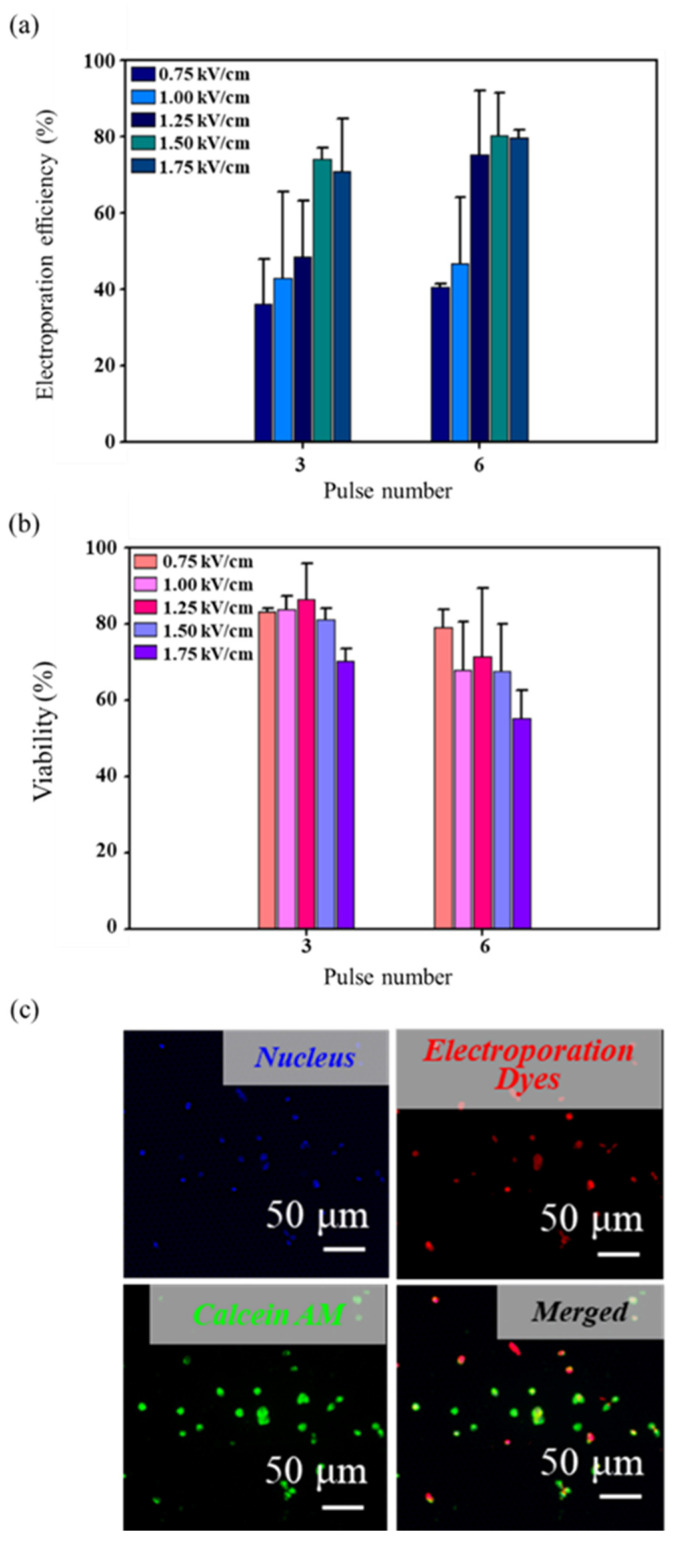
Transfection of fluorescent dyes in 1601 cells. (**a**) The electroporation efficiency of 1601 cells under different electric field strengths and pulse numbers (*n* = 3 for every trial). (**b**) The cell viability after electroporation under different electric field strengths and pulse numbers (*n* = 3 for every trial). (**c**) Typical fluorescent images of successful electroporation with viability maintained.

Figure 2a,b show that the electroporation efficiency inversely correlates with viability. When cells were exposed to electric field strengths higher than 1.25 kV/cm with six pulses, a high electroporation efficiency up to about 80% was exhibited, but the cell viability was less than 70%; whereas those electric field strengths of 0.75 kV/cm with three pulses showed a viability and electroporation efficiency of 83% and 36%, respectively. The optimum electric field strengths are required to consider viability and efficiency simultaneously. Thus, electric field strengths at 1.5 kV/cm, 100 μs pulse duration, three pulses, and 1000 ms pulse interval (denoted as 1.5 kV/cm/100 μs/3/1000 ms) is an optimal condition for both high electroporation efficiency (74.08 ± 2.94%) and cell viability (81.15 ± 3.04%). The short-term viability tested via the live/dead assay is shown in Figure 2c. Successful electroporation with maintained viability of 1601 cells showed triple-positive for blue, red, and green fluorescence colors (i.e., Nucleus+/EthD-1+/Calcein Green AM+).

**Figure 3:**
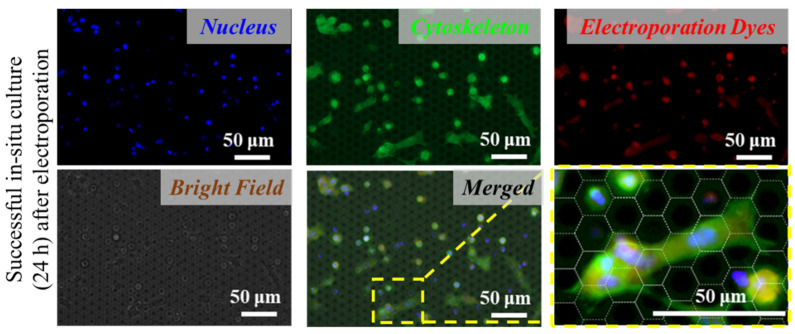
Good adhesion and spreading of the tumor cells on the PERFECT filter indicate successful in situ long-term (24 h) culture after electroporation.

In order to verify the long-term viability, in situ culture of the electroporated cells on the PERFECT filter was carried out. The well-presented adhesion, as shown in Figure 3, further confirmed the excellent performance of this in situ electroporation system. The compatibility for in situ culture and proliferation of presented systems may widen its applicability in downstream analyses, such as cell-based immunotherapy, gene-mediated therapy, and gene editing immunotherapy.

### 3.2. Plasmid Transfection

**Figure 4:**
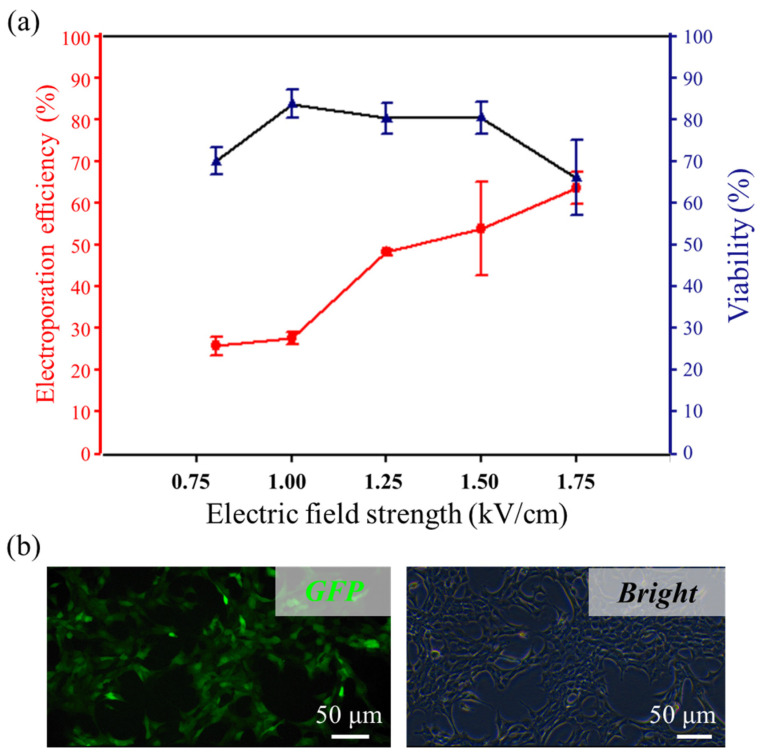
Transfection of plasmid in 1601 cells. (**a**) The electroporation efficiency and viability under different electric field strengths (*n* = 3 for every trial). (**b**) Fluorescence image of plasmid expression in the cells 24 h post-electroporation.

As a proof, taking the superior viability and electroporation efficiency of the proposed system, we conducted luciferase/GFP double reporter plasmid transfection of 1601 cells. Similar to the small molecule dyes experiment, a 10 mg/mL solution of plasmid was loaded and delivered into the separated 1601 cells using the in situ electroporation system. Under the identical electroporation condition (1.5 kV/cm/100 μs/3/1000 ms), the electroporation efficiency and cell viability were 54.07 ± 11.20% and 80.65 ± 3.74%, respectively, as shown in Figure 4a. A substantial reduction in transfection efficiency compared to the small molecule delivery could reflect the expected intracellular degradation and/or insufficient nuclear targeting of delivered plasmids after successful penetration across the cell membrane [43]. After delivery, the cells were incubated for another 48 h to allow for GFP expression, and the results were evaluated with fluorescence microscopy and quantitatively analyzed (Figure 4b). In situ manipulation of separation of tumor cells and the high viability of plasmid transfection made it possible to track CTCs for tumor metastasis study. However, for real CTC research, considering the extremely rare presence (1–10 cells/mL blood), imaging accuracy, and number of cells required for tumor formation need to be considered in further studies.

### 3.3. Specific Delivery of Functional Proteins

Identifying the tumor cells from a large number of background cells without cellular structure damage is a powerful strategy to monitor the targeted cells for the study of biological mechanisms. The electroporation-based method generally relies on the complex probe design [44], and traditional immunofluorescence (IF) staining is impossible to guarantee both high efficiency and high activity of cell labeling.

**Figure 5:**
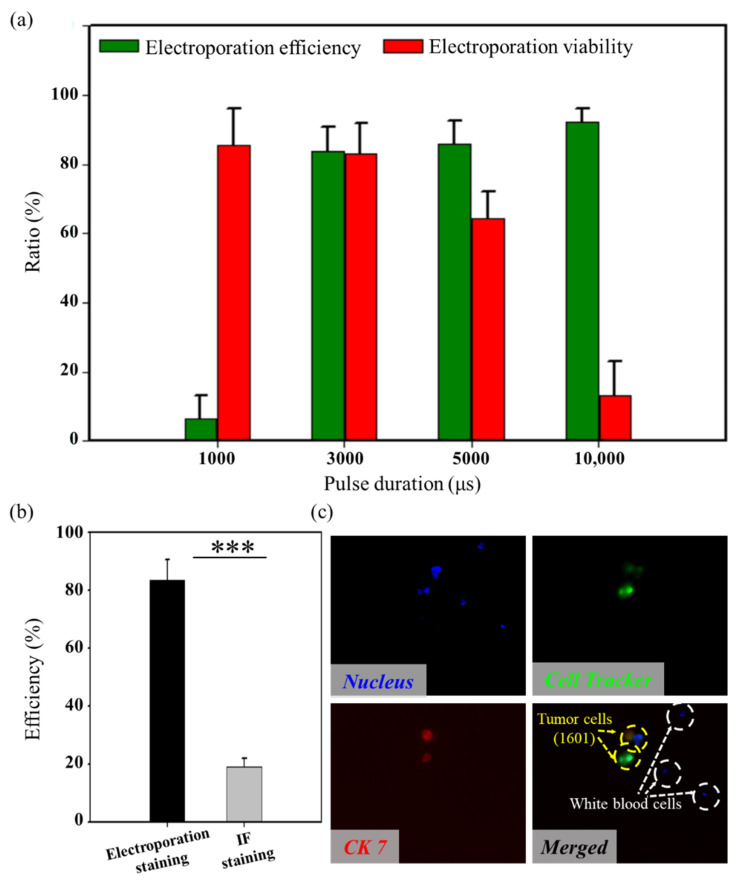
Transfection of dye-conjugated antibodies in 1601 cells. (**a**) The proteins electroporation efficiency and viability of 1601 cells under different pulse durations (*n* = 3 for every trial). (**b**) A comparison of transfection efficiency for the proposed system and the traditional IF staining (Wilcoxon rank-sum test, *** *p* < 0.001) (*n* = 3 for every trial). (**c**) Typical fluorescent images of tumor cell-specific electroporation.

We further explored the functional protein delivery using this in situ electroporation system for highly specific tumor cell labeling. Specifically, we established an antibody electroporation-based imaging approach to introduce fluorescent dye-conjugated antibodies into target living cells. The accumulation of biomolecules in the vicinity of the cell would cause diffusion-based delivery in the final stage [45], so the internal fluorescence intensities are directly proportional to the pulse durations. In order to achieve high-efficiency macromolecular protein delivery, four different pulse durations were used for testing. We observed that the variation of electroporation efficiency increased as the pulse duration increased. However, when the duration was 5000 μs, the cell viability was less than 80%, so we chose 3000 μs as an optimal condition (1.5 kV/cm/3000 μs/3/1000 ms), where the electroporation efficiency and cell viability were 83.7 ± 7.00% and 80.3 ± 8.71%, respectively (Figure 5a).

The cell labeling efficiencies obtained from the improved immunofluorescence staining were experimentally compared in parallel, as shown in Figure 5b. Highly specific cell labeling (98.6 ± 1.2%) was achieved (see Supplementary Material Figure S2), but the efficiency of the presented in situ electroporation system was significantly higher than the method of improved immunofluorescence staining. Within 10 min, the efficiency increased by 64.45%. Specific labeling of tumor cells from a large number of background cells (White blood cells, WBCs) was achieved, as shown in Figure 5c.

## 4. Conclusions

In this study, a high-efficiency in situ electroporation on the PERFECT filter platform is established for high viability and specific separating and labeling tumor cells from a large number of background cells with high throughput (7 × 10^5^ micropores per square centimeter). Three biomolecules (e.g., small molecule dyes, plasmids, and functional proteins) with different sizes were used to verify this in situ electroporation on the PERFECT filter system (the optimum electroporation parameters of three biomolecules are shown in Supplementary Material Table S1). By systematically varying electric field strengths and pulse numbers, high-efficiency small molecule dye and plasmid delivery were obtained. Cell viability after electroporation was confirmed by short-term staining, long-term in situ culture, and proliferation. In order to realize the protein delivery, a wider pulse duration was attempted. Fluorescent dye-conjugated antibodies were successfully introduced to tumor cells resulting in highly specific cell labeling. Overall, the high electroporation performances could be attributed to the combination of PERFECT filter-based tumor cell filtration and in situ electroporation. All-in-one operation reduces cell loss and damage between steps and controls the procedure operated in less than 10 min. In future work, a highly integrated dialysis system could be developed which allows for real CTC capture and labeling under dynamic electroporation on the PERFECT filter platform. Overall, we anticipate that this study was promised to be not only an efficient CTC separation and labeling technique but also a powerful tool to real-time monitor the activities of CTCs, which might provide valuable information for comprehensively understanding cancer pathogenesis and progression, and thus reveal new strategies for cancer diagnosis and tumor metastasis study.

## Supplementary Materials

The following supporting information can be downloaded at: https://www.mdpi.com/article/10.3390/mi13050672/s1. Figure S1: The separation efficiency and cell viability of 10,000 1601 cells in 1 mL PBS or whole blood, Figure S2: A comparison of transfection specificity for the proposed system and the traditional IF staining. Table S1: The optimum parameters of three types of molecules.
