# Supplementary material for: In Situ Electroporation on PERFECT Filter for High-Efficiency and High-Viability Tumor Cell Labeling
Source: Micromachines (Basel). 2022 Apr 26;13(5):672. doi: 10.3390/mi13050672 (PMC9146625; doi:10.3390/mi13050672)
Supplement: Supplementary file 1 [file micromachines-13-00672-s001.zip › micromachines-1672587-supplementary.pdf]

# In-Situ Electroporation on PERFECT Filter for High-efficiency and High-viability Tumor Cell Labeling

Tingting Hun[1], Yi Zhang[1], Qingmei Xu[1], Dong Huang[1, 2], Qi Wang[3], Zhihong Li[1, 2], Wei Wang[1, 2, 4]*

[1] School of integrated circuits, Peking University, Beijing, 100871, China
[2] National Key Laboratory of Science and Technology on Micro/Nano Fabrication, Beijing, 100871, China
[3] Department of Respiratory Medicine, the Second Affiliated Hospital of Dalian Medical University, Dalian, 116027, China
[4] Frontiers Science Center for Nano-optoelectronics, Peking University, Beijing, 100871, China
* Correspondence: w.wang@pku.edu.cn

Electronic Supplementary Information (ESI) available: Figure S1, Figure S2 and Table S1. See DOI:

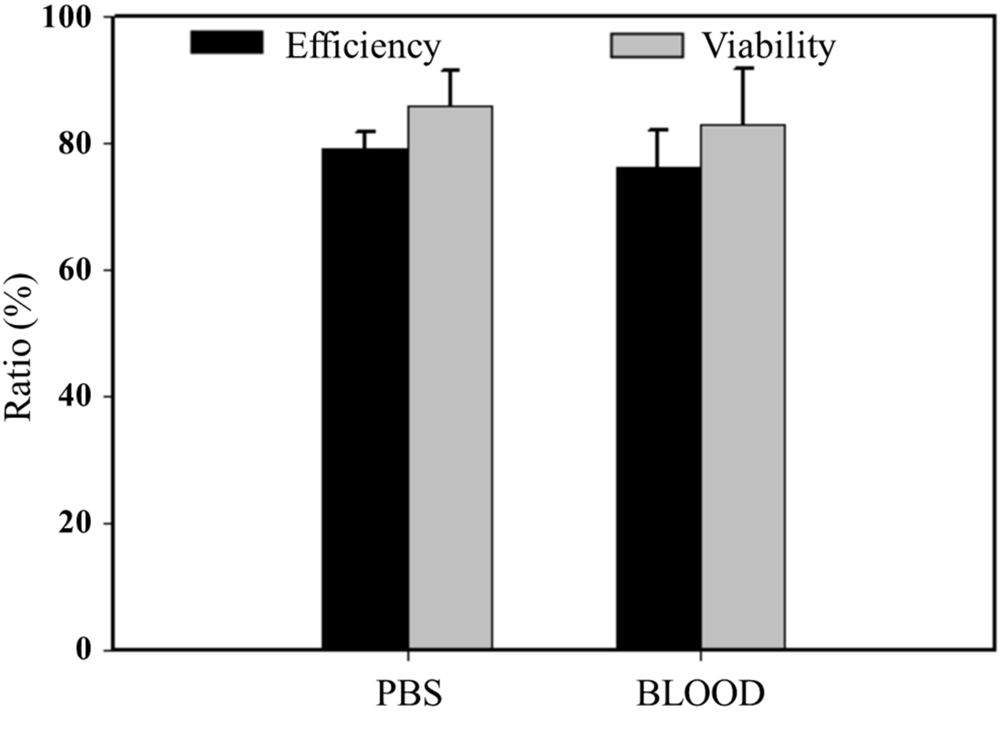


**Figure S1.** The separation efficiency and cell viability of 10000 1601 cells in 1 mL PBS or whole blood.

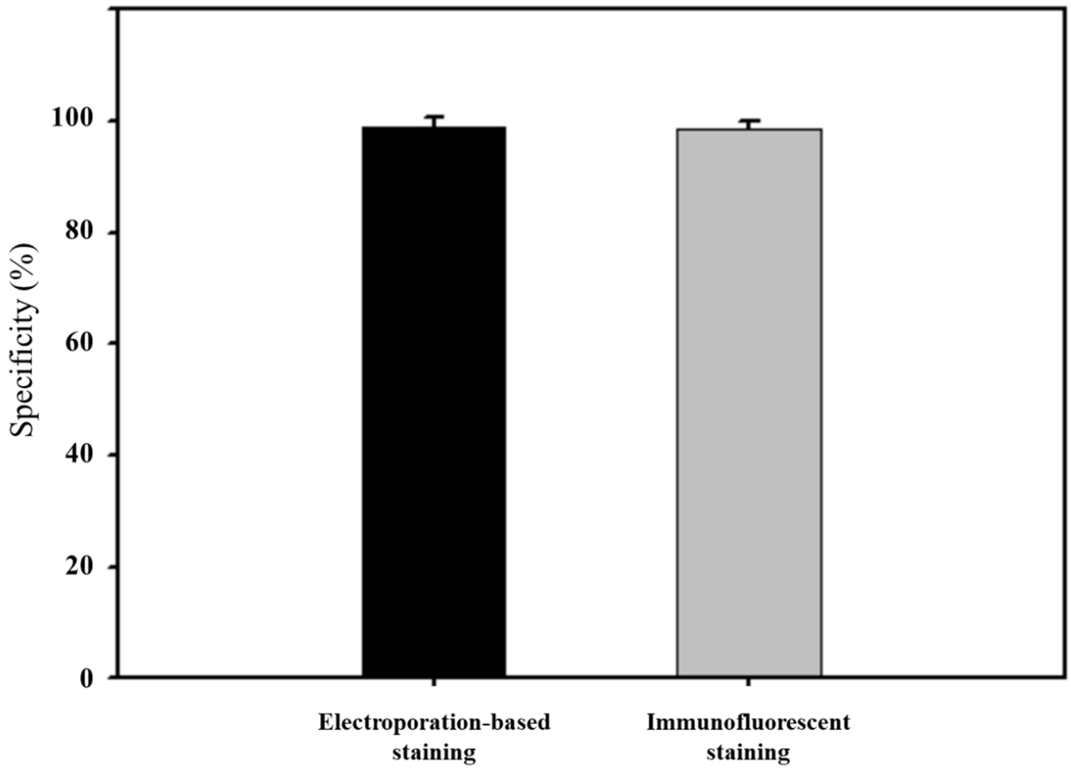


**Figure S2.** A comparison of transfection specificity for the proposed system and the traditional IF staining.

Table S1. The optimum parameters of three types of molecules

| **Type of molecules** | **Electric field strengths** | **Pulse duration** | **Pulses number** | **Pulse interval** |
|---|---|---|---|---|
| Small molecule dyes | 1.5 kV/cm | 100 μs | 3 | 1000 ms |
| Plasmid | 1.5 kV/cm | 100 μs | 3 | 1000 ms |
| Functional proteins | 1.5 kV/cm | 3000 μs | 3 | 1000 ms |
